# Galvanic Skin Response (GSR)/Electrodermal/Skin Conductance Biofeedback on Epilepsy: A Systematic Review and Meta-Analysis

**DOI:** 10.3389/fneur.2019.00377

**Published:** 2019-04-24

**Authors:** Yoko Nagai, Christopher Iain Jones, Arjune Sen

**Affiliations:** ^1^Brighton and Sussex Medical School, University of Sussex, Brighton, United Kingdom; ^2^Oxford Epilepsy Research Group, Nuffield Department of Clinical Neurosciences, NIHR Biomedical Research Centre, John Radcliffe Hospital, Oxford, United Kingdom

**Keywords:** epilepsy, biofeedback, galvanic skin response, skin conductance, electrodermal activity, autonomic activity, behavioral therapy

## Abstract

**Objectives:** Dynamic changes in psychophysiological arousal are directly expressed in the sympathetic innervation of the skin. This activity can be measured as tonic and phasic fluctuations in electrodermal activity [Galvanic Skin Response (GSR)/skin conductance]. Biofeedback training can enable an individual to gain voluntary control over this autonomic response and its central correlates. Theoretically, control of psychophysiological arousal may be harnessed as a therapy for epilepsy, to mitigate pre-ictal states. Evidence is accumulating for the clinical efficacy of GSR biofeedback training in the management of drug resistant epilepsy. In this review, we analyse current evidence of efficacy with GSR biofeedback and evaluate the methodology of each study.

**Method:** We searched published literature pertaining to interventional studies of GSR biofeedback for epilepsy, through MEDLINE and Cochrane databases (1950–2018). Using percentage seizure reduction as an indicator of therapeutic efficacy induced by GSR biofeedback, we used meta-analytic methods to summarize extant findings. We also compare and contrast study design with relevance to the interpretation of outcomes.

**Results:** Out of 21 articles retrieved for GSR/EDA/Skin conductance biofeedback, four studies were identified as interventional trials, involving 99 patients with drug-resistant epilepsy in total. Three of these studies included a control group and a positive therapeutic effect of biofeedback was reported in each of these. The difference in seizure frequency percentage (Biofeedback—Control) was between −54.4 and −74.0% with an overall weighted mean difference of −64.3% (95% CI: −85.4 to −43.2%). The response rates (proportion of patients manifesting >50% reduction in seizure frequency) varied from 45 to 66% across studies.

**Significance:** This timely evaluation highlights the potential value of GSR biofeedback therapy, and informs the optimal study design of larger scale studies that are now required to more definitively establish the utility of this non-invasive, non-pharmacological interventional approach for drug-resistant epilepsy.

## Introduction

Pharmacological therapy is the mainstay for the treatment of epilepsy. However, about 30% of people with epilepsy are drug-resistant and continue to have seizures despite optimal medications (1). There are alternative treatment options available for these patients, but these are limited to invasive and costly neurosurgical procedures including resection, Vagus Nerve Stimulation (VNS), Deep Brain Stimulation, and other options such as dietary modification. Biofeedback approaches offer non-invasive and likely cost-effective biobehavioral interventions. There is accumulating proof-of-concept evidence supporting biofeedback training as an efficacious means of reducing seizures in patients with drug resistant epilepsy (2–4).

Biofeedback training provides people with epilepsy a tool to learn how to voluntarily control usually-autonomous physiological signals: By providing conscious access to covert responses, an individual can learn to increase or decrease a physiological signal at will. Galvanic Skin Response (GSR) is an “electrodermal” signature of the sympathetic nervous innervation of the skin (5). GSR can be measured on the skin surface and predominantly reflects the unopposed action of sudomotor sympathetic nerves on secretory channels of eccrine sweat glands: enhanced porosity increases electrical conductance. GSR is familiar to many as the signal used in “lie detectors,” since GSR amplitude reacts sensitively to emotional provocation, salient thoughts, and attentional demand. Correspondingly, this effect exemplifies the direct coupling between sympathetic sweat gland innervation, measured by GSR, and brain states of affective and cognitive arousal.

### Development

Research into the effect of GSR biofeedback therapy on epilepsy was initiated by Nagai in 1997. The methodology was established through a series of neuroscientific studies, initially in healthy participants. The first published study, underpinning current GSR biofeedback methodology, described the relationship between peripheral autonomic activity and cortical excitation (6). Here, GSR amplitude was observed to inversely predict an encephalographic (EEG) index of cortical excitation; i.e., slow cortical potentials where there is a direct current (DC) shift within the EEG. Such DC shifts often precede epileptic seizures (7–9). Consequently, it was hypothesized that enhancement of peripheral sympathetic activity using GSR biofeedback may suppress the pre-ictal DC shift; this provided the rationale for the therapeutic use of GSR biofeedback training to reduce epileptic seizures (6).

The first clinical trial was conducted using a randomized controlled trial (RCT) design including a sham control (10). It is rarely possible to implement a full and effective double-blinding of behavioral (and cognitive) interventions without significantly compromising the logistics of delivering the therapy. Nevertheless, this study demonstrated a significant reduction in seizure frequency with a response rate of 60% controlling for non-specific effects of therapist contact. The efficacy of GSR biofeedback has since been replicated in three studies (11–13).

### Known Neural Mechanisms

The neural mechanisms through which GSR biofeedback might influence seizure thresholds were investigated in parallel with this first clinical trial. Three neuroimaging/EEG studies were conducted to characterize the detailed actions of GSR biofeedback on brain function. GSR biofeedback was observed to modulate activity across cortical and subcortical brain regions. Strikingly, however, activity within medial prefrontal cortex (MPFC) and orbitofrontal cortex (OFC) demonstrated a strong inverse correlation with tonic GSR (skin conductance levels) (14). These paralimbic cortices (MPFC and OFC) form a part of the default mode network; DMN (15). Abnormalities in functional activity and connectivity within the DMN have been reported in patients with epilepsy and associate with loss of consciousness during seizures (16, 17). Thus, repeated modulation of MPFC and OFC with GSR biofeedback training might influence the functional dynamics of this “consciousness” network.

A parallel neuroimaging study (18) also demonstrated that cortical arousal, reflected in the generation of slow cortical potentials during sensorimotor anticipation, is underpinned by activity within thalamus, cingulate cortex and supplementary motor area (SMA). Moreover, both healthy participants and patients with epilepsy demonstrate a reduced amplitude of (induced) slow cortical potentials after a course of GSR biofeedback training to increase sympathetic activity (6, 19). Together these studies linked changes in peripheral and central arousal to functional shifts within cortical and subcortical brain networks.

Lastly, a neuroimaging study in patients undergoing GSR biofeedback training confirmed that reduction of seizures is indeed linked to neural network changes involving the OFC (13): Here, GSR biofeedback training strengthened the functional connectivity between the OFC and the right amygdala. The degree of the functional connectivity changes was related to the reduction of patients' seizure frequency, such that patients who achieved greater right amygdala-OFC functional connectivity demonstrated a larger reduction in seizure frequency after 1 month of GSR biofeedback training. Psychological outcomes (reductions in anxiety and depression) did not account for either the seizure reduction (11–13), or functional connectivity changes (13). This indicates that changes in mental health states (such as anxiety and depression) are not necessarily direct contributing factors in reducing seizures. However, the positive correlation between increased fronto-limbic functional connectivity and degree of seizure reduction is worth further investigation. The amygdala and OFC form a physical white matter pathway, the uncinate fasciculus, thus repeated modulation of MPFC and OFC with GSR biofeedback training may also alter emotional cognition. Taken together these studies provide strong proof-of-concept support and validation for GSR biofeedback training as a promising approach to epilepsy management.

### Other Biofeedback Therapies in Epilepsy

The first application of biofeedback to epilepsy was in 1970 using EEG as a physiological parameter (20, 21). The feedback parameters of EEG biofeedback were broadly categorized into (1) enhancement of sensorimotor rhythms, which are considered to regulate thalamo-cortical circuitry (20), and (2) regulation of slow cortical potentials, which modulate cortical excitability (22). A meta-analysis of EEG biofeedback on epilepsy that included 10 studies reported more than 70% of participants achieved fewer seizures after EEG biofeedback training (4). The focus of this systematic review/meta-analysis is another type of biofeedback using GSR, the clinical effects of which appear more quickly following a less complicated procedure compared to EEG biofeedback.

### Objectives

In this systematic review, we compared studies from the perspectives of participant recruitment, intervention, outcomes, and study designs to evaluate the efficacy of GSR biofeedback therapy in reducing seizure frequency in people with drug resistant epilepsy. The objective of this article is also to draw together current evaluations of GSR biofeedback training efficacy to inform next-stage investigations and potential clinical introduction of a non-pharmacological intervention for drug resistant epilepsy. We also aim to guide optimal design of a future clinical trial.

## Materials and Methods

The methods used in this review follow the guidance of the PRISMA statement (2009) (23).

### Search Method and Data Collection

**Figure 1:**
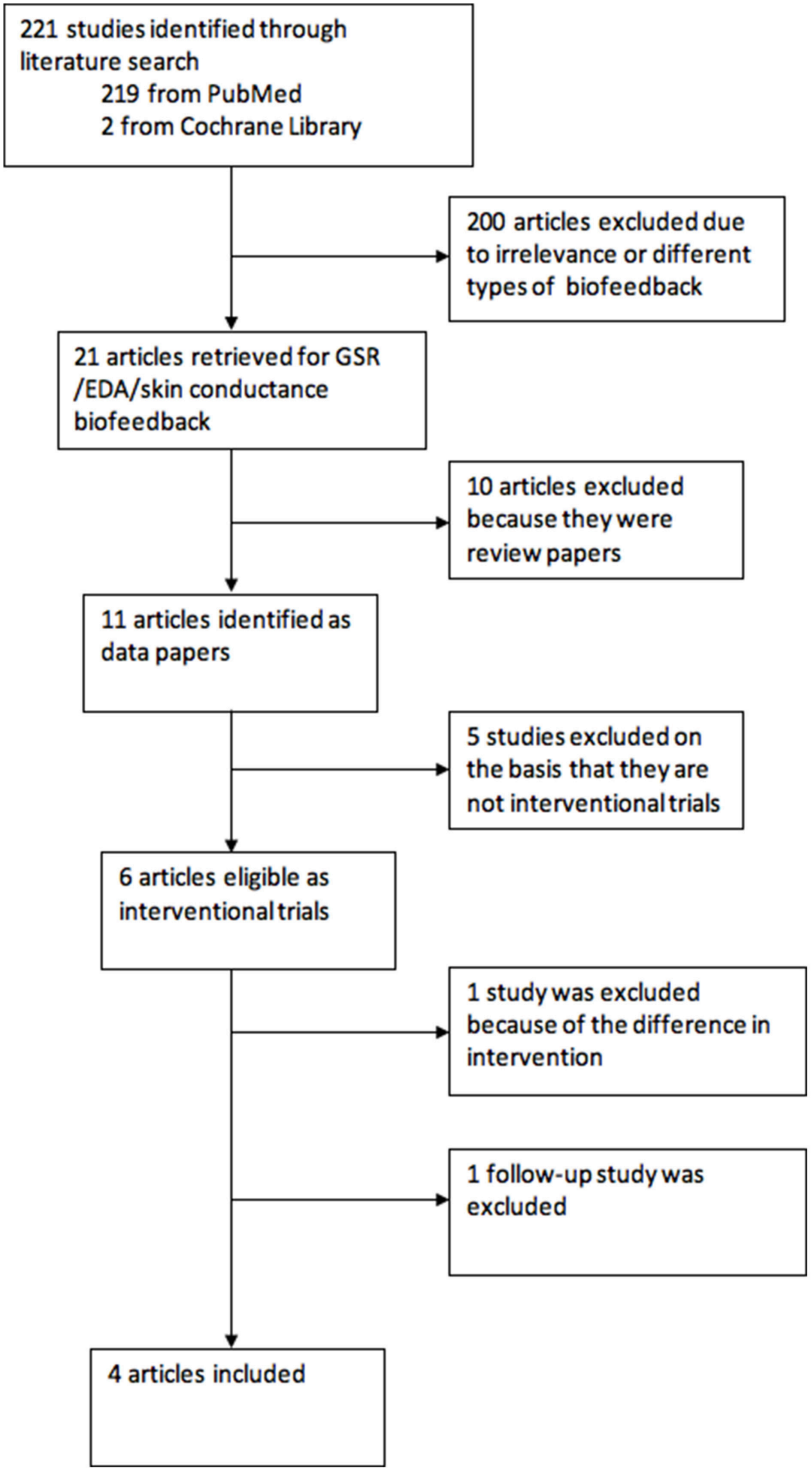
Flow chart of study selection.

We searched the published literature using Medline and Cochrane Library using the terms (“epilepsy” OR “seizures”) AND “biofeedback” AND (“galvanic skin response” OR “Electrodermal activity” OR “skin conductance”) published in English between 1st January 1950 and 31st July 2018. The article titles and abstracts were screened by YN and CIJ. The reports consisted of research papers, review articles and commentaries. The process of study selection is described in Figure 1.

### Inclusion and Exclusion Criteria

Only studies published in peer reviewed journals were selected. Articles were eligible for inclusion if they were interventional trials of GSR biofeedback to increase sympathetic activity. All non-interventional and follow-up studies were excluded. One study (24), exploring an effect of decreasing sympathetic activity, was also excluded from the formal analysis, however a description of the study is presented in the Table 1 for information.

**Table 1:** Study details.

**Study**	**Study design**	**Epilepsy types**	**No of patients**	**Age/gender**	**Protocol**	**Length of intervention**	**Outcome measures**
Nagai et al., (10)	RCT, single blinded, sham control	Varied	*N* = 21 B: 10 C: 8 Pilot: 3	16–60 M: 9 F: 9	Increase sympathetic activity	1 month	Seizure frequency, GSR change
Micoulaud-Franchi et al. (11)	Open study	TLE	*N* = 11	18–60 M: 2 F: 9	Increase sympathetic activity	3 months	Seizure frequency, GSR change, Psychological/cognitive measures
Scrimali et al. (24)	Case study	Brain malformation on right side	*N* = 1	NA	Decrease sympathetic activity	24 months	Seizure frequency,
Kotwas et al. (12)	Controlled, single blinded	TLE	*N* = 30 B: 15 C: 15	18–65 M: 10 F: 20	Increase sympathetic activity	3 months	Seizure frequency, GSR change, Psychological/cognitive measures
Nagai et al. (13)	Controlled, semi RCT, single blinded	TLE	*N* = 40 B: 20 C: 20	18–70 M: 16 F: 24	Increase sympathetic activity	1 month	Seizure frequency, fMRI (functional connectivity changes), Psychological measures

### Data Analysis

The primary outcome of interest in this review is the percentage difference in seizure frequency change between the control and biofeedback groups, where percentage seizure frequency change = (Post-averaged seizure frequency—baseline averaged seizure frequency)/baseline averaged seizure frequency × 100. The mean and SD of the change in seizure frequency for the individual control and biofeedback groups were collected from each study (in duplicate by CIJ and YN) and the weighted mean percent seizure frequency change was calculated individually for each group, using all available data. The weighted mean percentage difference, was then calculated for the studies that included both a control group and biofeedback group. Fixed effect models were fitted in Stata 15.1 using the metan command with the Mantel-Haenszel method. Fixed effects models were used due to the limited number of available studies. *I*^2^ was calculated where appropriate to estimate the percentage of variation that was due to heterogeneity between studies. In order to synthesize the evidence for the efficacy of the intervention, the study procedures of each articles were investigated in line with the PRISMA statement (23). The bias report was independently written by CIJ and reviewed by AS, neither of whom had conflicting interests in the study outcomes.

## Results

### Description of the Studies

#### Search Outcome

The electronic search found 221 articles. After eliminating 200 articles on the basis of relevance, 21 articles were left, of which 11 (52%) were research studies. Six studies were eventually selected as interventional studies, however one article was removed as the GSR biofeedback was provided with an atypical, instruction for biofeedback. Another study was a follow-up report on long-term effect of the intervention, which was again not analyzed but was included in discussion. This left four interventional studies of GSR biofeedback training in which the intervention was designed to increase sympathetic activity.

#### Participants

All studies investigated adult patients with drug resistant epilepsy (failure to respond to at least two appropriate anti-epileptic drugs). Three of the four studies focused on patients with temporal lobe epilepsy (TLE) (11–13). The average age of patients were similar, with slight differences reflecting differences in the age inclusion criteria. The mean baseline seizure frequencies were also similar between all studies. The gender ratio was not equal. Two studies recruited significantly more female, compared to male, patients. The brief characteristics of the participants from eligible studies are described in Table 1.

#### Study Designs

In chronological order, the first study (10) was a RCT, with a sham control condition. This study investigated 18 patients with drug resistant epilepsy (*n* = 10: therapy active group, *n* = 8: sham control group). The second (11) was an open-label study on 11 patients with TLE. The third study (12) was a controlled, non-randomized trial with 30 patients (*n* = 15: therapy active group, *n* = 15: treatment as usual group). The fourth (13) study was also a controlled trial with 40 patients with epilepsy (*n* = 20: therapy active group, *n* = 20: treatment as usual group) with partial randomization (deviation of 15%). These details are summarized in Table 1.

#### Therapy Protocols

All selected clinical trials delivered active therapy in the form of a face-to-face behavioral intervention. The pre- and post- seizure frequencies were recorded for 3 months in all trials. In two trials (10, 13), the intervention was provided in three sessions a week (each 30 min training duration), over 4 weeks. In contrast, the other studies (11, 12) provided 1 h sessions of the intervention once a week for 12 weeks. In all studies, GSR biofeedback was given to increase sympathetic activity. “Positive” visual feedback was given to indicate the desired direction of GSR amplitude change and was used by patients to voluntarily learn to control this index of alertness.

### Study Quality

#### Blinding

None of the studies were fully double-blinded, consistent with intrinsic difficulties in ensuring blindedness of behavioral therapies. However, key aspects of this problem were mitigated by allocating a follow-up assessor who was blinded to group membership in the last study (13).

#### Participants' Group Allocation

The first study (10) took the form of RCT, using a randomization table. The fourth study (13) also attempted full randomization as RCT, however this was not possible to achieve practically due to geographical considerations that affected logistics of patients' travel to the institution to receive behavioral therapy three times per week. Deviation from full randomization affected 15% of the participants. There is no mention of how participants were allocated in the third study (12), thus selection bias cannot be discounted.

#### Attrition

The first and fourth studies (10, 13) describe drop-out rates for patients and the data were analyzed using intention-to-treat analysis. There was no attrition reported in the other two studies.

### Meta-Analysis of Individual Study Results

#### Seizure Frequency Change

**Figure 2:**
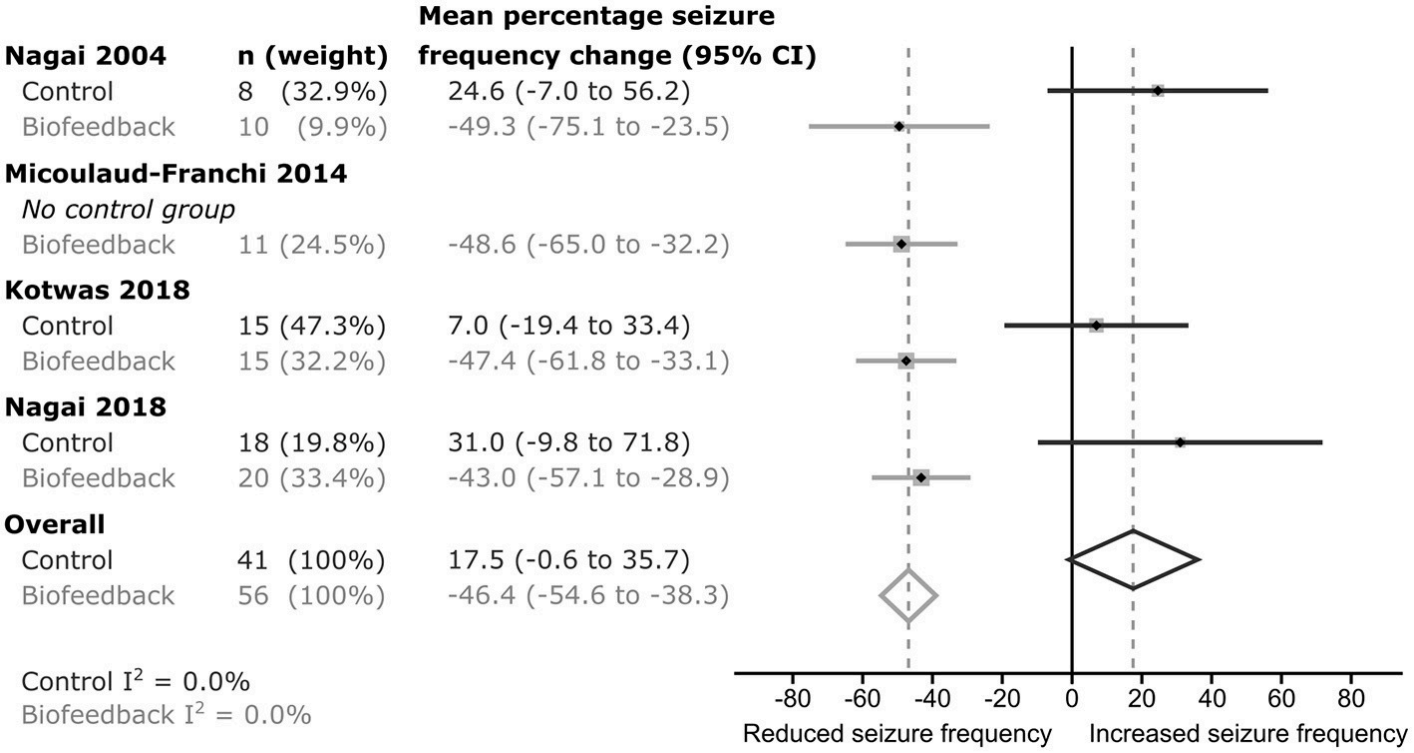
Forrest plot of the seizure frequency change within each group for each study. *I*^2^-values indicate there was low heterogeneity between studies.

**Figure 3:**
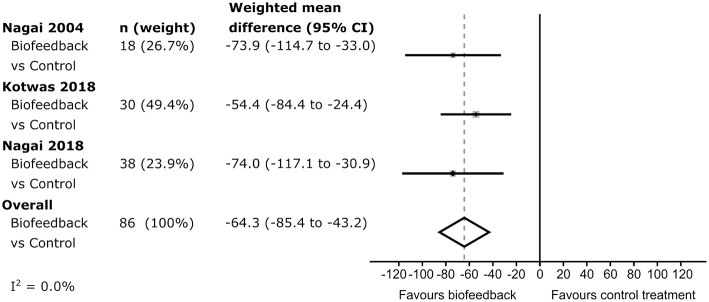
Forrest plot of the difference in seizure frequency change between control and intervention groups. The *I*^2^-value indicates there was low heterogeneity between studies.

In each of the studies, percentage seizure frequency change was calculated for each group from the patients' seizure frequencies (see methods), before and after control or biofeedback intervention. In the three studies including a control group, the average percentage frequency change in each control group was positive (i.e., more seizures), but all included 0 (no change) within their 95% confidence intervals (Figure 2). The weighted estimate of the percentage seizure frequency change across the control groups was 17.5% with 95% CI −0.6 to 35.7%, indicating no improvement (i.e., no reduction) in the patients' seizure frequency after receiving a control intervention. In each of the active biofeedback groups of the four studies, the percentage seizure frequency changes were negative (i.e., fewer seizures) with 0 outside their 95% confidence intervals. This mean reduction in seizure frequency was consistent between studies, between −43.0 and −49.3%. The weighted estimate across the four biofeedback groups was −46.4% with 95% CI −54.6 to −38.3%, indicating a reduction in seizure frequency for patients after receiving a biofeedback intervention (Figure 2). The weighted mean differences in seizure frequency change between the control and biofeedback groups in the three studies that included both groups were negative with 0 outside their 95% confidence intervals (Figure 3). This difference was consistent between studies, between −54.4 and −74.0%. The weighted mean difference across the three studies was −64.3% with 95% CI −85.4 to −43.2%, indicating a reduction in seizure frequency for patients receiving a biofeedback intervention compared to the control intervention. It is worth noting that there was an overall increased seizure frequency in the control group in all controlled studies, presumably reflecting natural fluctuation of seizure frequency.

#### GSR Change

The analysis of GSR was conducted in three of the four studies (10–12). Each study demonstrated a significant correlation between GSR amplitude and seizure frequency change: the range of correlation was between 0.54 and 0.74.

### Additional Analysis

Three studies (11–13) investigated alterations in secondary psychological outcomes and how these were related to changes in seizure frequency. Although there were observed changes in patient scores on psychological questionnaires measuring levels of anxiety and depression, no correlations were consistently found between these questionnaires and seizure frequency changes. Two studies (11, 12) also administered cognitive tasks to investigate the effect of the therapy on emotional processes by presenting emotional stimulation. However, no clear effect of the GSR biofeedback intervention was observed on cognitive outcomes, demonstrating no difference between therapy and control groups. One study (13) investigated functional neural connectivity changes before and after the GSR biofeedback. This study identified increased functional connectivity between the right amygdala and OFC was linked to reduction of seizure frequency.

## Discussion

This systematic review describes effects of GSR biofeedback on reducing epileptic seizures in patients with drug resistant epilepsy. Four interventional studies examined a total of 99 patients (56 in biofeedback groups). All studies demonstrated a reduction in seizure frequency through biofeedback training to increase sympathetic activity. This review also identified differences in study procedures that impact interpretation of the results as therapy outcomes and can inform the design of large-scale trials of the utility and cost effectiveness of the therapy in clinical settings.

### Summary of Evidence

The principal outcome of this systematic review is the support of evidence that GSR biofeedback training (to enhance sympathetic activity) is a potentially effective therapeutic tool to reduce epileptic seizures. Few studies have yet been conducted in this area, but of particular note was the consistency of the beneficial effect observed in patients with drug resistant epilepsy between studies (weighted estimate of −46.4% with 95% CI −54.6 to −38.3%). The magnitude of this effect is similar to that of other interventions for epilepsy, e.g., new anti-epileptic drugs 21–47% (25), VNS 30–70% (26) and ketogenic diets 30–55% (27). Despite the encouraging overall outcome of this non-invasive behavioral intervention, interpretations of the effect need to be made with caution, not least in consideration of the differences in design between studies. Out of three controlled trials, only one study was conducted using both a fully randomized and sham controlled design. All three trials with controls were single blinded. Thus, none of the studies can robustly eliminate the bias from expectation of therapists or patients. This is, however, a general problem for all behavioral therapies: blindedness is certainly one issue that has yet to be overcome when characterizing the core effect of GSR biofeedback therapy. Full randomization was attempted in two studies. However, this was modified in one study on logistical grounds. This highlights another difficulty associated with conducting pharmacological-style RCTs in behavioral therapies, since the study often demands that patients repeatedly travel to attend appointments for delivery of the active therapy in contrast to treatment-as-usual controls.

While the intervention impacted positively on seizure frequency, there was no correlation between seizure reduction and changes in patients' reported psychological states. In the trial that used neuroimaging (13), fronto-limbic connectivity changes predicted seizure reduction, however this functional connectivity changes were not correlated with changes in mood symptoms. Thus, the enhanced seizure control is putatively brought about by better integration of central arousal with visceromotor regulation (10) through a tonic modulation of amygdala—OFC coherence. However, these effects on therapeutic outcome were not mediated by subjective emotional states. This suggests that GSR biofeedback training can impact on pathological changes within the brain and low-level neural processes possibly supporting a core effect, rather than exerting therapeutic action such as a placebo response.

In comparison with the meta-analysis of EEG biofeedback, the GSR biofeedback studies were generally more robust than those on EEG biofeedback, where nine out of ten studies recruited fewer than 10 patients for open (non-controlled) interventions (4). The efficacies of GSR or EEG biofeedback appear similar however, as over 70% of participants demonstrated reduction of seizures in both meta-analyses. It is also worth mentioning that efficacy of GSR biofeedback can be observed as early as at 2 weeks of training, on the other hand, the standard training duration with EEG biofeedback is between 6 weeks and 6 months (3).

Overall, our literature analysis revealed the usefulness of GSR biofeedback in epilepsy. There is a paucity of therapeutic usage of GSR in clinical settings, although GSR is frequently used as a parameter of bodily arousal and increased attention in psychological studies. It is expected that this informative and easy to use autonomic parameter will be worthy of further investigation in terms of its interaction with central functions and with the other autonomic parameters.

### Limitations and Future Trial

The number of published studies using GSR biofeedback available for this systematic review was limited. Even compared to studies using electroencephalography biofeedback (neurofeedback), there is a paucity of studies utilizing (the more accessible) GSR biofeedback as a behavioral intervention. There is also little to indicate reporting bias, with unsuccessful applications going unpublished. Despite this limitation, this therapeutic approach appears promising. Future studies require more stringent and standardized study designs to affirm clinical efficacy in reducing epileptic seizures. Thus, at this stage, this systematic review provides valuable insight for planning such clinical trials. Across behavioral therapies, the issue of how to ensure blindness is a frequent focus of discussion for such trials. Similar issues apply to surgical interventions; since a clinician is actively responsible for delivering the intervention, this constrains the installation of double-blinded designs that are the gold-standard for pharmacological trials. However, technological advances can potentially circumvent this limitation. Digital technology with computerized and online platforms for the therapy may represent a viable alternative to face-to-face therapy that will not only permit double blinded studies, but may broaden access and reduce cost, enabling delivery of an effective non-drug treatment in pharmaco-resistant epilepsy.

## Conclusion

Despite the above limitations, this systematic review supports a view that modulation of sympathetic activity using GSR biofeedback represents a promising new therapeutic tool for management of seizures in patients with epilepsy. For future clinical trials, important elements to be considered include size of patient population, seizure type, double-blinding, inclusion of an appropriate control group, and robust randomization methods.

## Ethics Statement

We confirm that we have read the Journal's position on issues involved in ethical publication and affirm that this report is consistent with those guidelines.

## Author Contributions

YN is a pioneer of GSR biofeedback therapy for epilepsy and provided the systematic overview of historical development of this approach. YN wrote the first draft together with CIJ. CIJ is a medical statistician and was responsible for planning the statistical model and conducting the meta-analysis. AS provided independent oversight and unbiased expertise to the paper. All three authors contributed to the review, interpretation of the analysis, and writing of the paper.

### Conflict of Interest Statement

YN is named as the inventor on a patent for biofeedback treatment of epilepsy. YN has two related patents pending. YN is a director of a social enterprise: Biofeedback Global CIC (Community Interest Company) that aims to feed any financial gain back into the community in order to raise health care standards. The remaining authors declare that the research was conducted in the absence of any commercial or financial relationships that could be construed as a potential conflict of interest.

## Abbreviations

DMN: default mode network
EEG: electroencephalography
GSR: galvanic skin response
MPFC: medial prefrontal cortex
OFC: orbitofrontal cortex
TLE: temporal lobe epilepsy
VNS: vagus nerve stimulation.
